# Recent Advances in Imidazolium-Based Dicationic Ionic Liquids as Organocatalysts: A Mini-Review

**DOI:** 10.3390/ma15030866

**Published:** 2022-01-23

**Authors:** Fabiana Pandolfi, Martina Bortolami, Marta Feroci, Anastasia Fornari, Vincenzo Scarano, Daniele Rocco

**Affiliations:** Dipartimento di Scienze di Base e Applicate per l’Ingegneria (SBAI), Sapienza University of Rome, Via Castro Laurenziano, 7, I-00161 Rome, Italy; martina.bortolami@uniroma1.it (M.B.); marta.feroci@uniroma1.it (M.F.); anastasia.fornari@uniroma1.it (A.F.); vincenzo.scarano@uniroma1.it (V.S.)

**Keywords:** dicationic ionic liquids, organocatalysis, imidazolium DILs, solid supported DILs, organic synthesis

## Abstract

Imidazolium-based dicationic ionic liquids (DILs) are gaining considerable space in the field of organocatalysis mainly due to the opportunities in offering new possible applicable structural variations. In addition to the well-known variables which made the ionic liquids (ILs) famous as the type of cation and anion used, the nature of the molecular spacer moiety turns out a further possibility to improve some physicochemical properties, for example, solubility, acidity, electrochemical behavior, and so on. For this reason, this class of ionic liquids has been considered as possible competitors to their corresponding monocationic salts in replacing common catalysts in organic synthesis, particularly in cases in which their bidentate nature could positively affect the catalytic activity. This mini-review is intended to highlight the progress carried out in the last six years in the field of organocatalysis, including DILs as such and as hybrids with polymers, nanomaterials, and composites.

## 1. Introduction

Ionic liquids (ILs) have received increasing interest from the scientific community, due to their remarkable physicochemical properties (melting point, chemical and thermal stability, negligible vapor pressure, conductivity, solvating power, etc.) and their potential applications [1,2]. An ionic liquid is a salt which consists of a large organic cation associated to a non-coordinating organic or inorganic anion, melting at temperatures equal to or less than 100 °C. To date, several ILs are developed and they are often used as solvents, supporting electrolytes, catalysts, and in many other applications. ILs are considered, in most cases, green solvents, inert, easy to reuse, and with very low vapor pressure, reducing the possibility of air pollution, typical of common organic solvents. The wide choice of anions and cations gives the possibility to select the appropriate ionic liquid for a specific application [3]. In recent years, there has been an increased need to use organic synthesis methods that moderate the problem of environmental pollution. Green chemistry is dedicated to the optimization of synthetic procedures that minimize environmental impacts, both looking at a high atom economy (thus minimizing waste production, the use of polluting solvents, and non-stoichiometric reagents) and using reagents of low or no toxicity and persistence in the environment. For all these reasons, the attention shifted to the development of a new subclass of ILs called dicationic ionic liquids (DILs) because they demonstrated lower toxicity than corresponding monocationic ILs [4,5] and the possibility to widely modify their structures and thus their physicochemical properties [6]. In fact, the DILs contain two cationic heads connected by an aliphatic or an aromatic linker combined with two counteranions [7]. Consequently, some physicochemical properties, such as solubility, electrochemical behavior [7], viscosity, thermal stability, and others, can be modulated not only by varying the cation and the anion, but also the kind of linker between the two charged moieties [8,9,10,11]. DILs exhibit greater thermal and chemical stabilities, viscosity, flexibility, and lower volatility with respect to the corresponding monocationic ILs (see below). Moreover, the possibility to transform imidazolium ionic liquids (ILs and DILs) into the corresponding N-heterocyclic carbenes by electrochemical deprotonation, thus avoiding the use of any chemical base and the production of the corresponding byproducts [3], renders the use of these compounds even more attractive from the green chemistry point of view. For all these reasons, DILs show a growing interest in the field of catalysis. This mini-review is focused on the organocatalytic applications of imidazolium-based DILs, also associated with polymers, nanomaterials, and various composites reported in the literature in the last six years; this specific research field being very recent. To the best of our knowledge, only two recent reviews are reported in the literature about the properties and application of DILs; however, these documents do not specifically treat organocatalysis and do not extensively review the DILs use in organic synthesis from 2016 onwards [12,13]. We thus decided to start from these two reviews, reporting the results of DILs organocatalysis from 2016.

## 2. Applications in Organocatalysis

### 2.1. Imidazolium-Based Dicationic Ionic Liquids Used as Organocatalysts

In this paragraph, some examples of the use of imidazolium-based DILs as organocatalysts reported in the most recent literature are summarized and discussed.

The syntheses of DILs were previously reported and are thus not discussed in this mini-review [14,15,16,17,18,19,20,21,22].

**Scheme 1:**
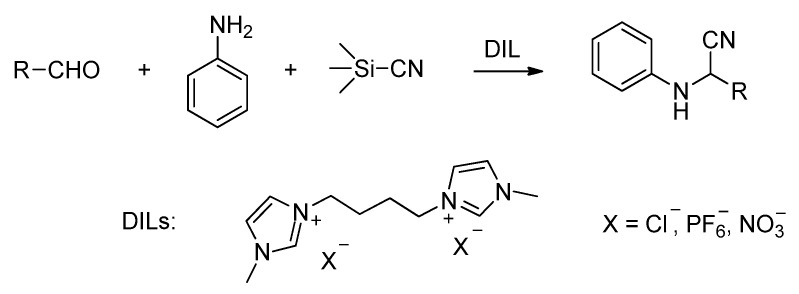
DILs catalyzed Strecker reaction: general reaction and structures of DILs used, reproduced with permission from [24].

Strecker reaction is one of the best known and investigated multicomponent reactions, which allows the synthesis of α-aminonitrile substrates through one-pot condensation of amine, carbonyl, and nitrile compounds [23]. Verma et al. used for the first time DILs as catalysts in the Strecker synthesis starting from trimethylsilylcyanide (TMSCN), aniline, and differently substituted benzaldehydes (Scheme 1) [24].

The experimental results were compared with those obtained using monocationic ILs and higher catalytic activity was observed in favor of DILs (97% reaction yield vs. 73% using the same wt%). The authors attributed this result to the bifunctional nature of the diimidazolium salts.

**Scheme 2:**
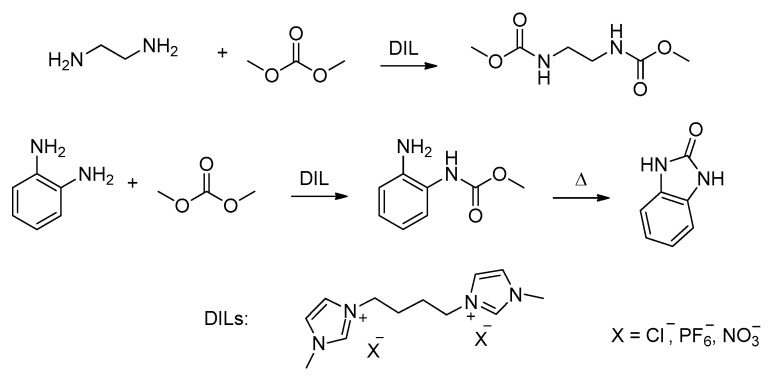
DILs catalyzed the synthesis of carbamate, reproduced with permission from [25].

The same authors also reported the application of these DILs as catalysts in the selective synthesis of carbamates, starting from a diamine and using dimethylcarbonate as a carboxylating agent, without solvent [25]. The reaction was performed for the carboxylation of an aliphatic (ethylenediamine) and an aromatic (*o*-phenylenediamine) amine with dimethyl carbonate (Scheme 2).

The reaction conditions were optimized by studying the effect of the temperature and the catalytic dosage, in order to reduce the formation of byproducts, a recurrent problem in this kind of reaction (methylated amine and monocarbamate).

Even in this case, the yields using DILs as catalysts were higher than those obtained in the presence of monocationic ILs, probably due to the double cationic moieties. The hypothesized mechanism suggests that the C-2 proton of imidazolium cationic part improves the polarization of the dimethylcarbonate carbonyl group by a hydrogen bonding interaction, facilitating the amine nucleophilic attack. Moreover, the role of the counterion is to activate the amine, therefore the catalytic activity increases simultaneously with the H-bond acceptor ability of DILs anions. Among them, the maximum yield was achieved with the DIL in which the counterion was PF_6_^−^ (93% and 88% starting from aliphatic and aromatic diamine, respectively). The authors also extended the study to other amine substrates with good results. 

**Scheme 3:**
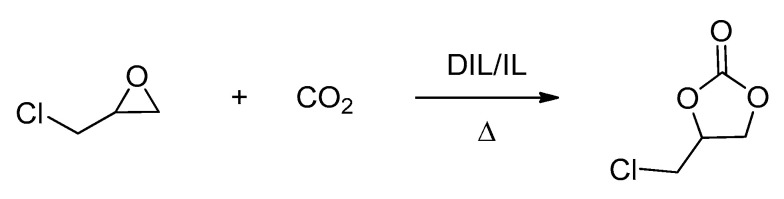
DILs and ILs catalyzed cycloaddition of CO_2_ to epichlorohydrin, reproduced with permission from [30].

**Figure 1:**
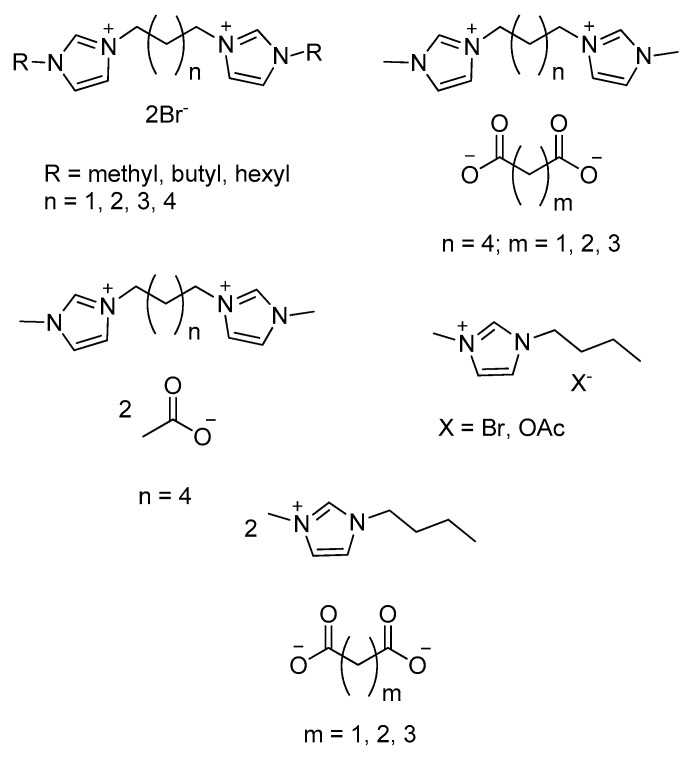
Structures of the studied DILs and ILs by Guglielmero et al., reproduced with permission from [30].

The increase in carbon dioxide amounts in the atmosphere causes serious problems for the environment, mainly due to air pollution and the consequent global warming [26,27]. Many efforts have been made by researchers to find strategies for alternative CO_2_ utilization, such as the capture, storage, and transformation of CO_2_ in order to minimize these problems [28,29]. In the last years, several articles focused on this issue and in these cases, DILs organocatalysis proved to be useful for the cycloaddition of CO_2_ to epoxide derivatives. The catalytic activity of the imidazolium-based ILs in this kind of reaction is manifested by the acidic H-2 on the imidazole ring, which activates the epoxide (epichlorohydrin as substrate), but at the same time, it could decrease the nucleophilicity of the chloride. In 2019, Guglielmero et al. evaluated the ability of some DILs, diversified by the alkyl group on the imidazole ring, the length of the alkylene spacer, and the type of the counterion in the cycloaddition of CO_2_ to epichlorohydrin (Scheme 3), comparing them with the corresponding monoimidazolium ILs (Figure 1) [30].

The researchers studied the influence of the temperature (from 60 °C to 100 °C) for the series of methyl imidazolium bromide DILs, using 1 mol% of catalyst, CO_2_ at 10 bar, carrying out the reaction for two hours. In general, the reaction yields increased with the temperature, while the pressure of CO_2_ did not seem to be a determinant. In order to evaluate if the trend of the yield with the increase in the temperature was due to the solubility of the catalyst in the reaction medium, this property was measured for all bromide salts. Although the solubility increased with the increase in the alkylene (oligomethylene) spacer chains, all DILs were not totally soluble even at 100 °C, unlike the corresponding monocationic ILs, which were completely soluble in epichlorohydrin, also at lower temperatures. This behavior was ascribed to the dicationic nature and to the extensive hydrogen-bond interactions between the anion and the hydrogen atoms of the imidazolium cation. The problem of a not complete dissolution was overcome by adding a 20 mol% of the reaction product. Regarding the other studied DILs, with fixed reaction conditions (80 °C, 1 mol% of catalyst, 10 bar of CO_2_, 2 h), the authors observed that the DILs substituted with imidazole rings with a methyl or a hexyl alkyl chain showed higher catalytic activity with respect to DILs with a butyl chain. Moreover, the efficiency of the catalysis increased with the increase in the length of the linker, except for the butyl series in which an opposite trend was detected. Finally, mono- and dicarboxylate (D)ILs as catalysts resulted in a lower catalytic activity compared with bromide (D)ILs.

**Figure 2:**
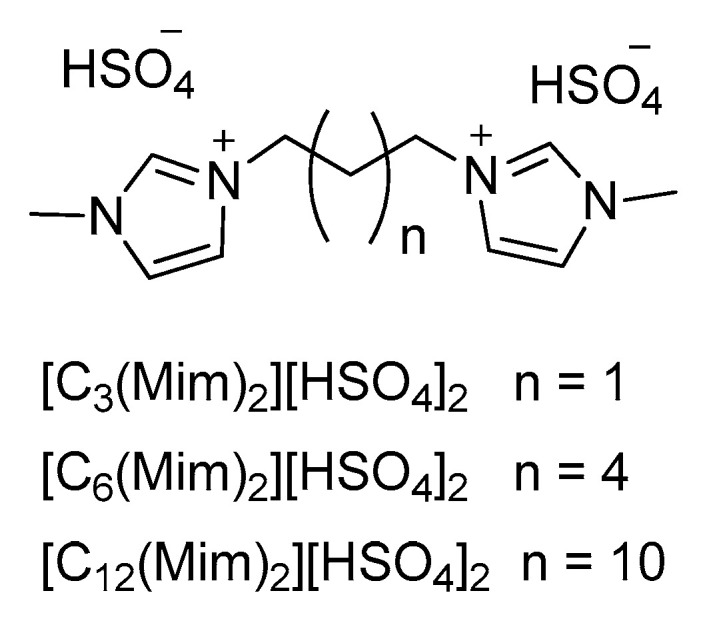
Structures of the BrØnsted acidic DILs studied by Yang et al., reproduced with permission from [31].

Yang et al. exploited the BrØnsted acidity of imidazolium-based DILs to catalyze the esterification of long-chain free fatty acids with methanol. The authors prepared three different BrØnsted acidic DILs, bearing three ([C_3_(Mim)_2_][HSO_4_]_2_), six ([C_6_(Mim)_2_][HSO_4_]_2_) and twelve ([C_12_(Mim)_2_][HSO_4_]_2_) methylene groups as alkyl spacer chains (Figure 2), to evaluate their catalytic activity in the esterification reaction of oleic acid and methanol.

All three synthesized DILs showed good performances since the expected product of the reaction, oleic acid methyl ester, was obtained with excellent yields (>90%). The experimental results revealed a close relationship between the catalytic activity of DILs and the spacer chain length because the catalytic performances increased with the diminution of the distance between the two cationic heads. This trend is in accordance with the DILs’ acidity, which decreases with the elongation of the alkyl spacer [31].

Moreover, Zekri et al. prepared two novel highly acidic imidazolium-based DILs with HSO_4_^−^ as counterion. The authors tested these catalysts for the esterification reaction of phthalic anhydride with different types of alcohols, obtaining the corresponding phthalates with higher final yields than those obtained with conventional catalysts, using a lower amount of alcohol and a shorter reaction time [32].

**Scheme 4:**
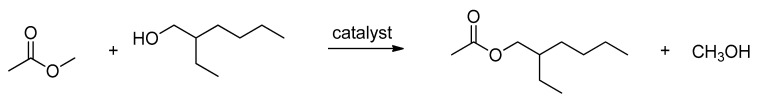
DAHPA-ILs catalyzed transesterification reaction of methyl acetate with isooctyl alcohol, adapted from [33].

Liu et al. synthesized two novel acidic heteropolyanion-based DILs (DAHPA-ILs) to be applied as catalysts in the transesterification reaction of methyl acetate with isooctyl alcohol (Scheme 4) [33].

The authors chose HPW_12_O_40_^2−^ as anion moiety varying the cationic one, using in one of the two cases the imidazolium head [Bis-BsImB][HPW_12_O_40_]. This novel catalyst showed much higher catalytic activity than the corresponding monocationic salt [MIM]_3_PW_12_O_40_ with a conversion of methyl acetate obtained by 53.36% and 1.96%, respectively. In addition, the catalytic performances of [Bis-BsImB][HPW_12_O_40_] were comparable with those of conventional acidic catalysts, H_2_SO_4_ (55.47%) and H_3_PW_12_O_40_ (55.04%), with the disadvantage, however, of the latter being a homogeneous catalyst and therefore difficult to separate from the reaction mixtures.

**Figure 3:**
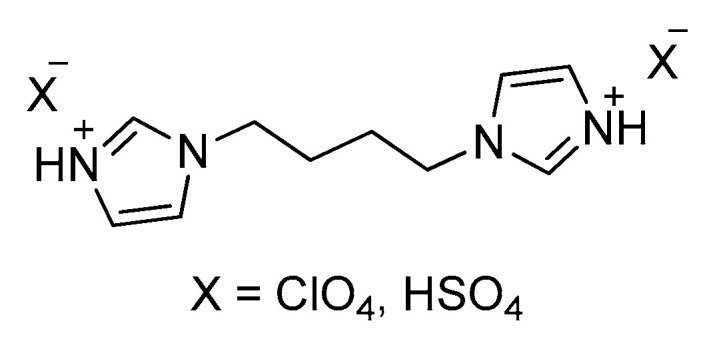
Structures of BrØnsted acidic DILs studied by Daneshvar et al., reproduced with permission from [34].

Daneshvar et al. developed two BrØnsted acidic DILs (Figure 3) to catalyze the synthesis of recurring moieties in compounds with biological and pharmaceutical activities: 5-arylidene barbituric acids and pyrano [2,3-*d*] pyrimidinone derivatives [34].

**Scheme 5:**
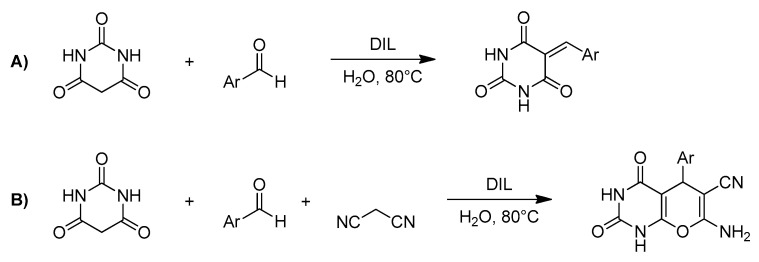
Bronsted acidic DILs catalyzed the synthesis of 5-arylidene barbituric acids (**A**) and pyrano[2,3-*d*] pyrimidinone derivatives (**B**), reproduced with permission from [34].

As represented in Scheme 5, pyrimidine-2,4,6(1*H*,3*H*,5*H*)-trione reacted with variously substituted aryl aldehydes by a Knoevenagel condensation to obtain 5-arylidene barbituric acids (Reaction A) or, in the presence of malononitrile, by a multicomponent reaction, to synthesize pyrano[2,3-*d*] pyrimidinone derivatives (Reaction B).

The catalytic activity of these DILs was tested using a wide range of aldehydes, achieving the desired products with excellent yields. Finally, the authors demonstrated that the catalysts could be reused in up to five reaction cycles.

**Figure 4:**
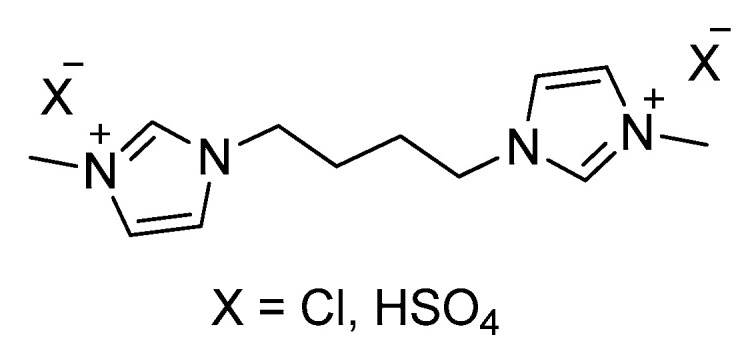
Structures of N-methyl imidazolium-based DILs studied by Daneshvar et al., adapted from [35].

**Scheme 6:**
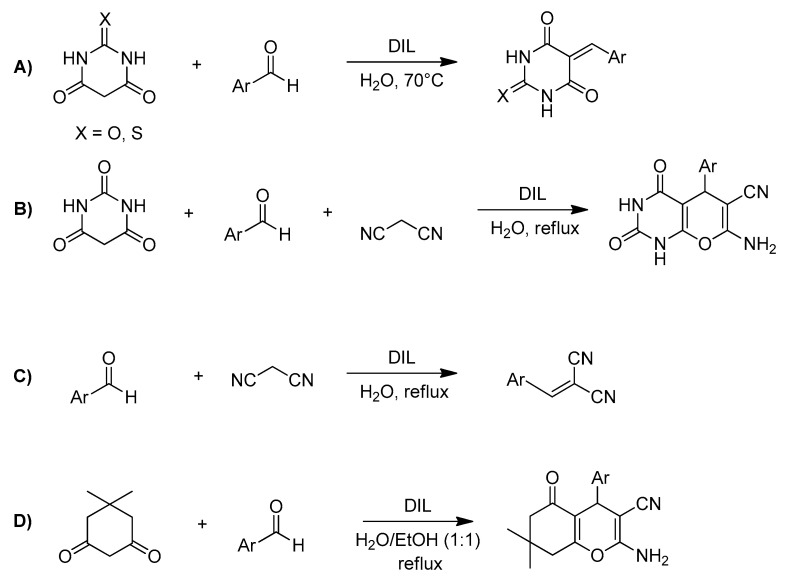
DILs catalyzed of 5-arylidene (thio)barbituric acids (**A**), pyrano[2,3-*d*] pyrimidinone derivatives (**B**), 2-arylidene malononitriles (**C**), and 4*H*-pyrans (**D**), adapted from [35].

Later, the same authors synthesized two *N*-methyl imidazolium-based DILs (Figure 4) with the aim to catalyze similar reactions, represented in Scheme 6 [35].

They synthesized 5-arylidene (thio)barbituric acids and 2-arylidene malononitriles, derived from Knoevenagel condensation, and 4*H*-pyrans and pyrano[2,3-*d*]pyrimidinones with good yields. The two catalysts acted differently. In particular, the DIL containing hydrogen sulfate as counterion was not able to catalyze the synthesis of 4*H*-pyrans, and in general, the DIL with chloride as counterion demonstrated the best efficiencies when the reactions needed a basic or weak acidic environment. On the contrary, bis hydrogen sulfate DIL worked better when the reaction required acidic media.

Imidazolium-based DILs have been used as catalysts to reduce the disparity of the reactivity of the endo-OH and exo-OH of isosorbide (ISO), a bio-based monomer employed as an alternative to bisphenol A (BPA) in the synthesis of polycarbonates. This difference in reactivity is one of the main obstacles for obtaining poly-(isosorbide carbonate) (PIC) with high molecular weight, a fundamental requirement of engineering plastics.

**Figure 5:**
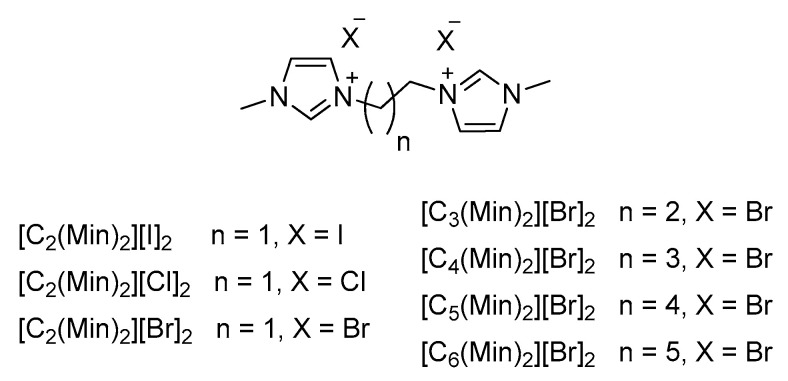
Structures of DILs reported by Wang et al., reproduced with permission from [36].

**Scheme 7:**
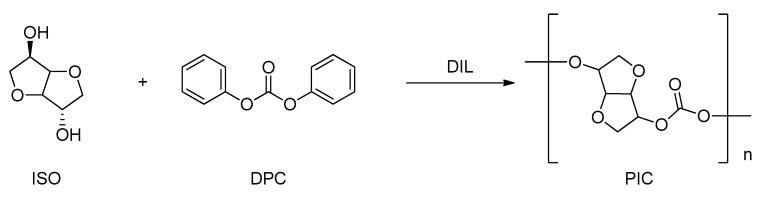
DILs catalyzed the synthesis of poly (isosorbide carbonate), reproduced with permission from [36].

Wang et al. synthesized a series of DILs including halogen anions (Figure 5) as organocatalysts to increase, at the same time, the reactivity of the endo-OH and the carbonyl group of diphenyl carbonate (DPC) in order to synthesize high molecular weight PICs (Scheme 7).

The authors investigated the influence of the structure of DILs on the synthesis of PIC, by observation of experimental results and by DFT calculations. In particular, the increase in cationic head spacing led to lower catalytic activity of DILs, obtaining molecular weights from 89,800 g mol^−1^ for [C_2_(Min)_2_][Br]_2_ to 74,100 g mol^−1^ for [C_6_(Min)_2_][Br]_2_. Moreover, DFT progressive calculations highlighted the weakening of the interaction energy between the imidazolium cations and the DPC carbonyl group as a response to the increase in the number of the spacer methylene units, resulting in a decrease in the catalytic activity. [C_2_(Min)_2_][Br]_2_ showed better performances even compared with its structural analogous with having different anions ([C_2_(Min)_2_][Cl]_2_ and [C_2_(Min)_2_][I]_2_). The increase in electronegativity (Cl^−^ > Br^−^ > I^−^) favors the interaction between the anion and the hydroxyl group of ISO, but at the same time the increase in the anionic ray results in a weaker ionic pair, meaning a greater ability of the anion to release itself from the cation and interact with the substrate. The experimental results showed that the bromide ion is the best compromise between these two experimental issues [36].

**Scheme 8:**
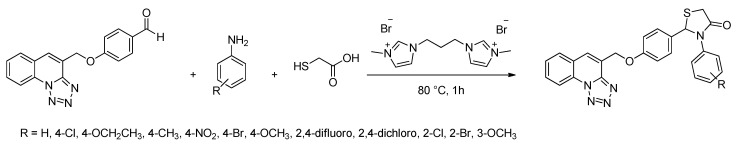
[C_3_(Min)_2_][Br]_2_ catalyzed one-pot cyclocondensation of 4-(tetrazolo[1,5-*a*]quinolin-4-ylmethoxy)benzaldehyde, anilines, and mercapto acetic acid to obtain 3-substituted phenyl-2-(4-(tetrazolo[1,5-*a*]quinolin-4-ylmethoxy)phenyl)thiazolidin-4-ones, adapted from [37].

Deshmukh et al. used the DIL [C_3_(Min)_2_][Br]_2_ to catalyze a one-pot cyclocondensation of 4-(tetrazolo[1,5-*a*]quinolin-4-ylmethoxy)benzaldehyde, anilines, and mercapto acetic acid to obtain 3-substituted phenyl-2-(4-(tetrazolo[1,5-*a*]quinolin-4-ylmethoxy)phenyl)thiazolidin-4-ones as antitubercular compounds (Scheme 8) [37].

Previously, the same authors synthesized this class of molecules in PEG-400, but the reaction mixture was heated at 110 °C for 2 h, obtaining the desired compounds with modest final yields [38]. Instead, the DIL organocatalyst allowed to improve the reaction conditions (80 °C for 1 h) with excellent yields (81–92%).

Overall, the experimental results obtained to date highlighted a higher catalytic activity of DILs with respect to ILs, most probably due to the bidentate moiety. Moreover, the spacer length seems of paramount importance with regard to the catalytic activity, although a uniform trend cannot be drawn, and each reaction type needs an optimization study. It is quite astonishing that no paper reported the use of a spacer containing a chemical function which could enter the reaction mechanism; we strongly suggest future studies explore this field.

### 2.2. Imidazolium-Based Dicationic Ionic Liquids Composites and/or Immobilized on Solid Support as Organocatalysts

Frequently, it is possible to find in the literature some organocatalysis applications of imidazolium-based DILs as composites and/or immobilized on solid supports such as silica, polymers, metal ions, zeolites, and many others, with the aim of making them more applicable to industrial processes.

As an example, graphene oxide was frequently used as an ionic liquid support for many applications [39,40].

Patel et al. reported the catalytic activity of graphene-oxide-supported -SO_3_H functionalized imidazolium-based DIL (DIL@GO) in some one-pot multicomponent reactions to synthesize compounds of great interest in medicinal chemistry [41,42]. The catalyst DIL@GO was carefully characterized by X-ray diffraction (XRD) and thermogravimetric analysis (TGA), scanning electron microscopy (SEM), transmission electron microscopy (TEM), Fourier-transform infrared spectroscopy (FT-IR), energy dispersive X-ray spectroscopy (EDX), and elemental analysis.

**Scheme 9:**
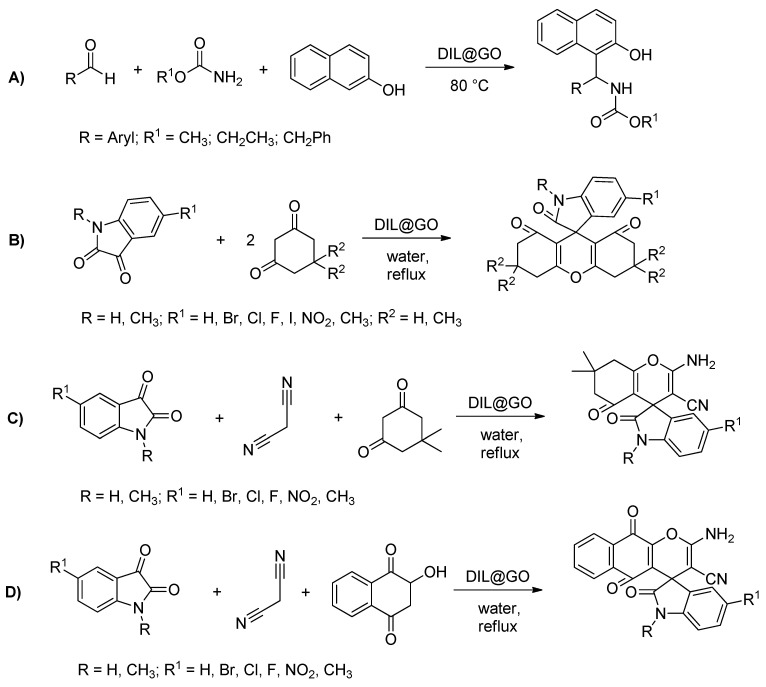
(**A**) Synthesis of 1-carbamatoalkyl-2-naphthols using DIL@GO; (**B**) synthesis of spiro[indoline-3, 9′-xanthene]trione derivatives; (**C**) synthesis of spiro[chromene-4,3′-indoline]-3-carbonitrile derivatives using dimedone; and (**D**) synthesis of spiro[chromene-4,3′-indoline]-3-carbonitrile derivatives using 2-hydroxynaphthalene-1,4-dione, adapted from [41,42].

In particular, they synthesized 1-carbamatoalkyl-2-naphthols derivatives from aromatic aldehydes, 2-naphthols, and alkyl carbamates in neat conditions (Scheme 9A); spiro[indoline-3,9′-xanthene]trione derivatives from substituted isatins and 1,3-diketone compounds (1,3-cyclohexanedione/dimedone) (Scheme 9B); and spiro[chromene-4,3′-indoline]-3-carbonitrile derivatives from substituted isatins, malononitrile, and enolizable C-H-activated compounds, dimedone (Scheme 9C) or 2-hydroxynaphthalene-1,4-dione (Scheme 9D).

The syntheses were optimized by varying the catalyst amount and the reaction temperature; all compounds were obtained with a >90% yield and a reaction mechanism was suggested. Finally, the authors demonstrated the remarkable advantages of the use of this catalyst, such as the good stability, the easy work-up and separation from the reaction medium, and the reusability of up to five reaction cycles without a significant drop in its activity.

**Scheme 10:**
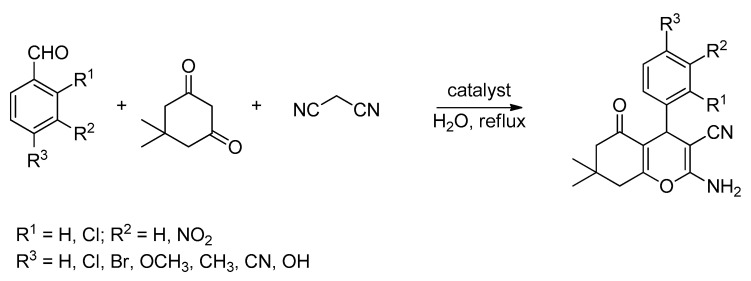
Synthesis of 4*H*-chromene derivatives catalyzed by an imidazolium-based DIL stabilized on Fe_3_O_4_ and SiO_2_ magnetic nanoparticles, reproduced with permission from [43].

Hormozinezhad et al. developed a new imidazolium-based DIL catalyst stabilized on Fe_3_O_4_ and SiO_2_ magnetic nanoparticles with MnCl_4_^2−^ as counterions, describing the synthesis and the morphological and chemical characterizations [43]. The authors tested this catalyst in a one-pot three-component condensation reaction for the synthesis of 4*H*-chromene derivatives starting from substituted aromatic aldehydes, malononitrile, and dimedone (Scheme 10). After the optimization of the reaction conditions, the best yields (85–92%) were obtained using water as the solvent under reflux in short reaction times (15 min). Moreover, the catalyst was easily reusable by removing it through the application of an external magnetic field.

**Scheme 11:**
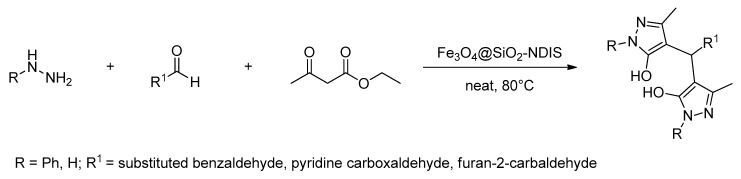
Fe_3_O_4_@SiO_2_-NDIS catalyzed the synthesis of bis-pyrazole-5-ols from various aldehydes, phenylhydrazine/hydrazine hydrate, and ethyl acetoacetate, reproduced with permission from [44].

Some compounds with remarkable pharmaceutical activities contain bispyrazole moiety, which is commonly synthesized by a one-pot reaction starting from phenylhydrazine, ethyl acetoacetate, and aryl aldehydes, in presence of a catalyst. Often this reaction shows many disadvantages, such as long reaction time, the necessity of toxic organic solvents, and the non-recoverability of the catalyst. In order to improve the synthesis, an interesting approach was developed by Rezaei et al. [44]. In particular, the researchers designed, prepared, and characterized a new catalyst based on a Brønsted acidic DIL immobilized on silica-coated iron oxide support nanoparticles (Fe_3_O_4_@SiO_2_-NDIS) and they evaluated its catalytic activity in the synthesis of 4,4’-(arylmethylene)bis(3-methyl-1H-pyrazol-5-ol)s. The effective immobilization of silica and DIL on iron oxide was confirmed by FT-IR. The shape and the diameter of the nanoparticles were determined by SEM, TEM, and dynamic light scattering analysis (DLS), resulting in spherical nanoparticles, in which the dark magnetic core was encapsulated into lighter amorphous silica shells, with a diameter of 100–150 nm or 200 nm. The ζ-potential value was −73.5 mV for nanoparticles in absence of DIL and became positive after the introduction of the DIL, which modified the surface of nanoparticles with acidic sites. Having magnetic properties, the catalyst was easily removed from the reaction environment by an external magnetic field. The reaction conditions were optimized on a reaction model and the best yield was obtained in neat conditions at 80 °C. Then, the general applicability of these reaction conditions was confirmed by carrying out the one-pot condensation between a series of aromatic aldehydes (electron-withdrawing and electron-donating benzaldehyde, pyridine carboxaldehyde, or furan-2-carbaldehyde), ethyl acetoacetate, and phenylhydrazine or hydrazine (Scheme 11). In all cases, excellent yields were achieved.

Finally, the recovery of the catalyst by applying an external magnetic field was demonstrated in up to five reaction cycles and the recovered catalyst was characterized again by SEM and TGA techniques, showing no significant changes compared with the fresh catalyst.

The literature reports that structural modifications imposed on cyclodextrins (CDs) can lead to considerable advantages in their application as catalysts.

**Scheme 12:**
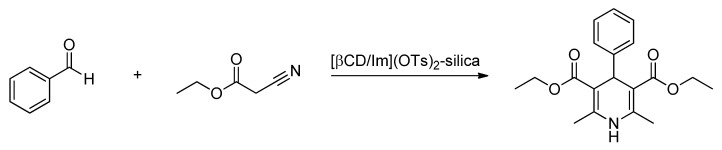
[βCD/Im](OTs)_2_-silica composite catalyzed the synthesis of diethyl 2,6-dimethyl-4-phenyl-1,4-dihydropyridine-3,5-dicarboxylate starting from benzaldehyde and ethyl 2-cyanoacetate, adapted from [45].

In view of this, Moheiseni et al. incorporated CDs moieties with imidazolium-based DIL and supported it on silica gel, in order to increase their ability to complex different types of guest molecules [45]. The authors tested the catalytic activity of this CDs/ionic liquid hybrid system [βCD/Im](OTs)_2_-silica in the synthesis of 1,4-dihydropyridine and polyhydroquinoline derivatives starting from arylaldehydes, β-ketoesters, and nitrogen donors, for example, as ammonium acetate (Hantzsch reaction). Over the years, numerous and different protocols of this reaction have been developed, under different reaction conditions and using many types of catalysts, nevertheless, several disadvantages are common to some of these reactions, such as harsh experimental conditions, low yields of the final products, high catalyst loading, and long reaction times. Instead, the [βCD/Im](OTs)_2_-silica composite led to the development of a new Hantzsch reaction synthetic procedure with objective benefits regarding the reaction times (15 min, in the experiment which showed the best experimental conditions) with significant results of final product yield (98%, Scheme 12).

The Glaser heterocoupling reaction is one of the most used procedures for the synthesis of unsymmetrical 1,3-diynes; however, it is affected by some drawbacks, such as the homocoupling byproducts, the high cost, and the poor recyclability of the catalysts commonly used. In fact, starting from an alkynol and phenylacetylene, one of the alkyne precursors needs to be in excess to avoid a homocoupling reaction and to obtain a high yield of the heterocoupling product. For all these reasons, Lai et al. developed a new catalyst consisting of lignosulfonate/DIL composite as a support for preparing a heterogeneous Cu-based catalyst (DIL@LS-TMEDA-Cu) [46]. Their strategy was to prepare a hydrogen-bond-active solid supporting material to allow a locally high concentration of the alkynol, in order to use a low substrate ratio of reactants, but at the same time, being a phenylacetylene a lipophilic molecule, the support should not be too hydrophilic. Thus, they functionalized sodium lignin sulfonate with a DIL to produce a hydrophobic polymer, which is also able to establish hydrogen bonds, using it as a supporting material to immobilize a copper-tetramethylethylenediamine (Cu-TMEDA) catalyst for the first time.

**Scheme 13:**
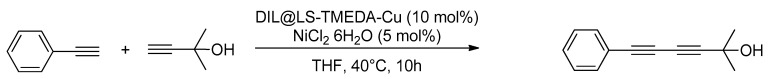
DIL@LS-TMEDA-Cu catalyzed heterocoupling reaction of phenylacetylene and alkynol, reproduced with permission from [46].

The success in obtaining this material was confirmed by elemental analysis and FT-IR spectroscopy and the loading of Cu by inductively coupled plasma mass spectrometry (ICP-MS). The morphology and the composition of this support were studied by means of X-ray photoelectron spectroscopy (XPS), SEM, and TEM analyses. Moreover, the material showed higher thermostability below 300 °C. The authors, carrying out a model reaction, found that the optimal conditions were to be as follows: 10 mol % of DIL@LS-TMEDA-Cu, 5 mol % of NiCl_2_ 6H_2_O, 1.0 equivalent of Et_3_N, THF as solvent, 40 °C, and 10 h. To guarantee a good yield and to avoid a high substrate ratio of reactants, the reactions were performed with a ratio of 2 to 1 of phenylacetylene and alkynol, respectively (Scheme 13).

Extending the optimized conditions to other substrates (phenylacetylenes with different substituents and various types of alkynols), generally good yields were achieved. Differently, low yields were obtained starting from 4-fluorophenylacetylene, probably due to H−F type of hydrogen bond that could be established with the support. Finally, the catalyst was recovered up to four times without loss of its activity.

**Scheme 14:**
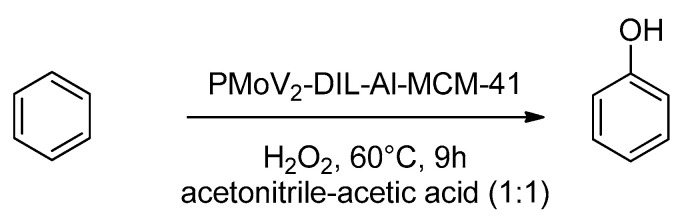
Selective oxidation of benzene to phenol by PMoV_2_-DIL-Al-MCM-41, reproduced with permission from [47].

Taheri et al. synthesized an organic–inorganic composite of anchored DIL phosphovanadomolybdate anions on mesoporous aluminosilicate (PMoV_2_-IL-Al-MCM-41) as catalyst for selective oxidation of benzene to phenol, using H_2_O_2_ as the oxidant (Scheme 14) [47].

The structure of the synthesized hybrid was confirmed by various techniques. The authors found the best conditions to carry out the selective oxidation of benzene to phenol by analyzing the reaction mixture with HPLC, changing various parameters: the amount of catalyst, temperature, molar ratio of H_2_O_2_ with respect to benzene, reaction time, and solvent used. The best conditions were a molar ratio of H_2_O_2_/benzene of 4:1, a temperature of 60 °C, nine hours of reaction time, and a mixture of acetonitrile and acetic acid with a volume ratio of 1:1 as solvent. The recovery of the organic–inorganic composite showed after five runs a decrease in the reaction yields from 14.8% to 6.3%, but retained 100% of the selectivity.

**Scheme 15:**
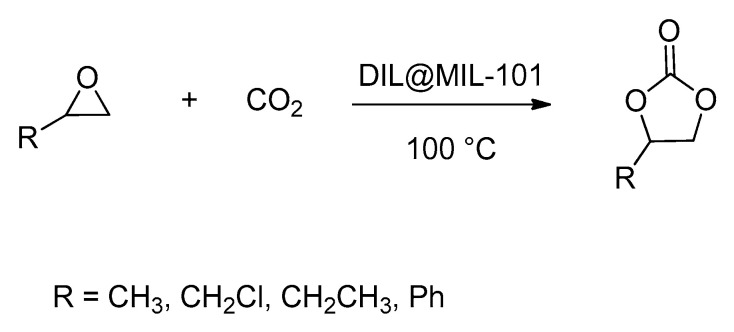
DIL@MIL-101 catalyzed the cycloaddition of CO_2_ on epoxides, reproduced with permission from [48].

As reported in a previous paragraph, the CO_2_ cycloaddition to epoxides is an important issue for green chemistry. Jiang et al. used metal–organic frameworks (MOF) chromium-benzenedicarboxylate (MIL-101) to immobilize an imidazolium-based DIL bromide and they studied the resulting catalyst (DIL@MIL-101) on this kind of reaction [48]. Other MOFs and IL/MOFs composites were previously investigated in the literature, but their synthesis was too complicated and expensive. The authors developed a new easier synthesis for DIL@MIL-101 via a ship-in-bottle technique and, after a full morphological and chemical characterization, they evaluated its catalytic activity on the reactions reported in Scheme 15, obtaining good yields.

Finally, the researchers tested the reusability of this catalyst up to three times, without any loss of its catalytic activity.

**Figure 6:**
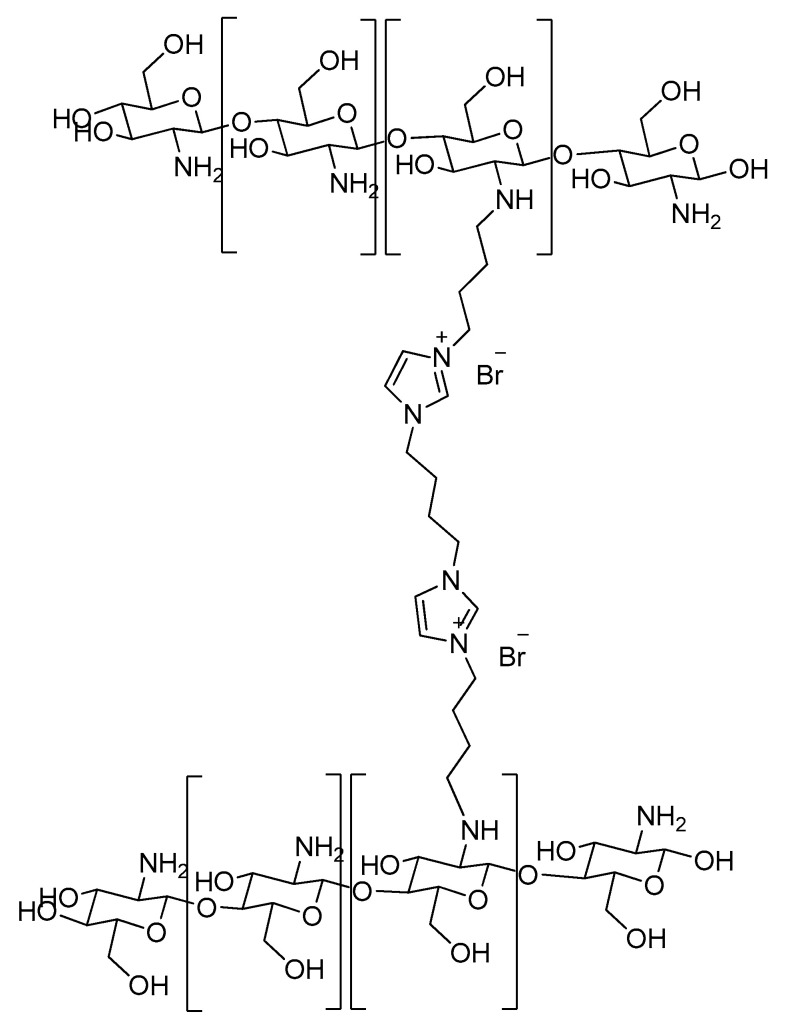
Chitosan/DIL composite structure, reproduced with permission from [49].

In 2018, Taheri et al. synthesized a chitosan/DIL composite (Figure 6), with the aim of catalyzing the CO_2_ cycloaddition to various epoxides to obtain cyclic carbonates [49]. An additional positive note is given by the fact that the authors used chitosan, a cheap and renewable biopolymer.

The catalyst was characterized by various techniques. A model reaction was selected to optimize the reaction conditions (120 °C, 20 bar of CO_2_ pressure, 5 h, without solvent), that were then extended to the same epoxides seen in Scheme 15. Under the optimized conditions, other DILs were synthesized by anion exchange of bromide with chloride and carbonate anion and their catalytic activities decreased as expected.

**Figure 7:**
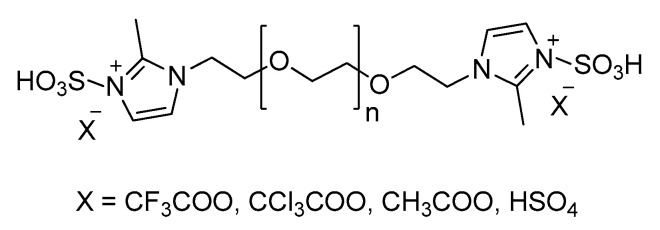
Structures of PEG-6000 bridged DILs, adapted from [50].

Gogoi and Borah reported the synthesis of a series of imidazolium-based DILs containing N-sulfonic cationic heads, in order to improve the BrØnsted acidic properties, associated with four counterions, CF_3_COO^−^, CCl_3_COO^−^, AcO^−^, and HSO_4_^−^ (Figure 7) [50]. The two cationic moieties were linked with polyethylene glycol (PEG-6000), chosen for its known important characteristics, such as temperature-dependent phase behavior in solution, eco-compatibility, water solubility, and thermal stability.

**Scheme 16:**
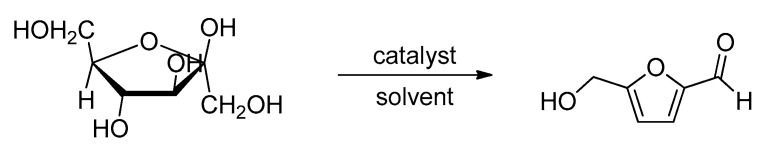
Conversion of fructose into HMF, adapted from [50].

The acidity of these DILs was measured using UV–Vis spectroscopy to generate the Hammett plots. The TGA showed that all DILs were thermally stable up to 150 °C. The authors tested the catalytic activity of this material in the conversion of fructose to 5-hydroxymethyl furfural (HMF), an important reaction in biomass transformation to obtain renewable resources, in several organic solvents (Scheme 16) [51,52].

The HMF was quantified by HPLC analysis, using a C_18_-reverse-phased column equipped with a UV detector. In particular, in all cases, the 100% conversion was achieved in acetonitrile or in ethyl acetate, varying the reaction time (6 h or 12 h). Nevertheless, the acidities of the DILs were not in accordance with the percentage conversion of the fructose. Therefore, the authors hypothesized that the major reasons of the catalytic activity were accounted to the ion-pair strength of DILs and to their interaction with the fructose.

Later, Prasad et al. developed a heterogeneous catalyst in which the preparation of ZSM-5 zeolite was assisted by a DIL as template material, in order to study its activity on the selective conversion of fructose into HMF [53]. The researchers compared this newly synthesized catalyst with the commercial ZSM-5 zeolite, with regard to both morphology and catalytic activity. These two materials showed identical results by FT-IR and were diversified in the surface area and the porosity. The reaction was carried out in 1-butyl-3-methylimidazolium chloride (BMImCl) as the solvent, at 110 °C for 90 min. The commercial ZSM-5 and the synthesized DIL-assisted ZSM-5 zeolite showed 68% fructose conversion (46.2% HMF yield) and 92% conversion (47.2% HMF yield), respectively. The synthesized material was also compared with some commonly used catalysts (FeCl_3_ 6H_2_O, CuCl_2_ 2H_2_O, CoCl_2_ 6H_2_O), demonstrating in all cases better efficiencies.

**Figure 8:**
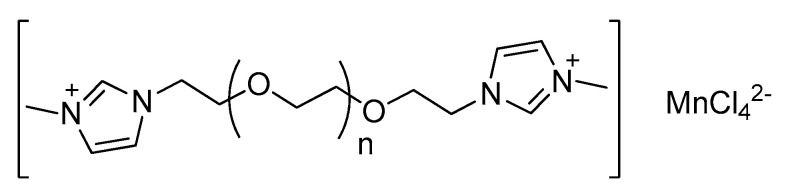
Structure of PEG-DIL-based MnCl_4_^2−^ used as phase transfer catalyst, reproduced with permission from [54].

Benzyl azides and thiocyanates are common intermediates useful in the synthesis of compounds with biological activities and with industrial interest. Generally, these molecules are synthesized by nucleophilic substitution of aryl halides in a multiphase environment. With the aim to perform this reaction in water from the perspective of green chemistry, Goodajdar et al. developed a PEG-DIL-based MnCl_4_^2−^ (Figure 8) as a phase transfer catalyst [54].

**Scheme 17:**
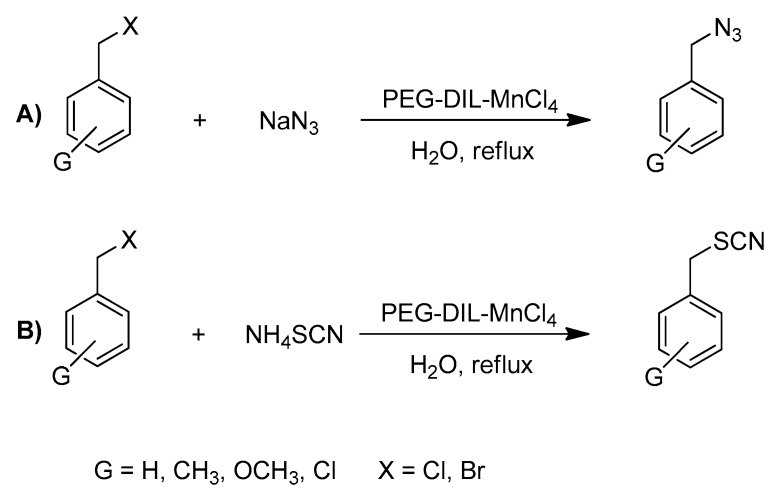
PEG-DIL-MnCl_4_ as phase transfer catalyst for the synthesis of benzyl azides (**A**) and thiocyanates (**B**), reproduced with permission from [54].

The catalyst was characterized by FT-IR, UV–Vis and Raman spectroscopies and the latter demonstrated the presence of MnCl_4_^2−^ in the structure of DIL. Moreover, TGA curves showed thermal stability up to 250 °C. After the optimization of the reaction conditions on a model reaction (100 °C, water as solvent), the researchers tested the general applicability of this catalyst in the reaction of a variety of benzyl halides with sodium azide (Scheme 17A) and with ammonium thiocyanate (Scheme 17B). Excellent yields were achieved in all cases, both with benzyl halides bearing electron-donating and electron-withdrawing substituents. Instead, regarding alkyl halides, a good yield was obtained with a primary halide while, probably due to steric hindrance problems, the secondary halide did not react even after five hours.

**Figure 9:**
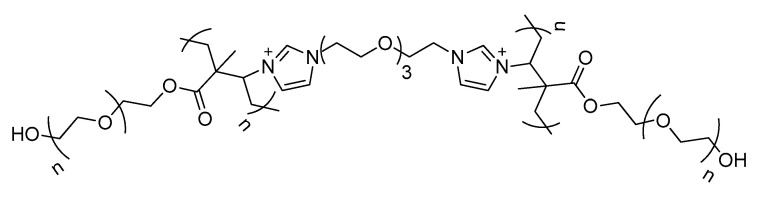
Imidazolium-based DIL synthesized by Reddy et al., reproduced with permission from [55].

Reddy et al. synthesized an imidazolium-based DIL in which the two cationic heads were linked by tetra-ethylene glycol and the nitrogen contained a polyethylene glycol methacrylate moiety as substituent, the mesylate anions were chosen as counterions (Figure 9) [55].

**Scheme 18:**
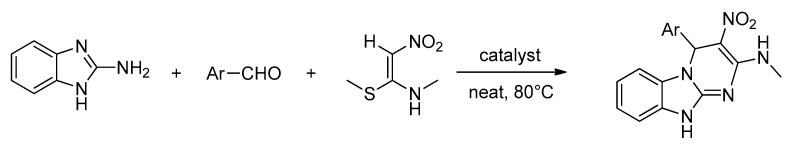
*N*-methyl-2-nitro-aryl-benzo[4,5]imidazo[1,2-a]pyrimidine amines catalyzed by polyethylene glycol methacrylate-grafted tetra-ethylene glycol-bridged imidazolium-based DIL, reproduced with permission from [55].

The authors demonstrated the excellent catalytic activity of this DIL in a multicomponent reaction starting from 1*H*-benzo[d]imidazol-2-amine, (*E*)-*N*-methyl-1-(methylthio)-2-nitroethenamine, and a wide selection of aromatic aldehydes for the synthesis of the corresponding *N*-methyl-2-nitro-aryl-benzo[4,5]imidazo[1,2-a]pyrimidine amines in neat conditions (Scheme 18).

The products were obtained in excellent yields (90–96%) and the catalyst was recovered up to seven reaction cycles. This work provided a useful protocol to obtain compounds with biological activities in environmentally friendly conditions.

Although the addition of at least a chemical step is necessary, the immobilization of DILs over solid supports renders their use as organocatalysts more suitable for industrial applications, due to the ease in catalyst recovery and recycling. Moreover, when the support is magnetic, the removal of the catalyst is even easier (external magnetic field), adding a higher attractiveness to this kind of catalyst. Nonetheless, it cannot be excluded as having a role in the support in the studied reaction, and possibly the choice of the solid support could be intentionally done in view of such a role. Lastly, due to the lack of papers reporting the application of solid-supported DILs in flow chemistry (a greener way of conducting organic synthesis), we strongly suggest exploring this research field in the future.

## 3. Conclusions

In this mini-review we summarized a series of reactions in which imidazolium-based DILs are used as organocatalysts in their unaltered state or as composites and/or immobilized on solid supports (Table 1).

In both cases, this class of DILs has demonstrated all their potential and opportunities which can offer in the field of organic synthesis, resulting mainly from the higher number of structural variables that can be applied if compared with their corresponding monocationic salts. Preserving the majority of the physicochemical properties of ILs, DILs allow to modulate and control other properties such as acidity or solubility in the most common organic solvents, not only by the type of cationic heads and counterion chosen but also by the spacer molecular portion. Moreover, the authors have often explained the higher catalytic activity of imidazolium-based DILs, with respect to the corresponding monocationic ILs, with the bifunctional nature of salts, especially in the reactions in which the catalytic activity is mainly concentrated on cationic heads. Precisely for this reason, this class of DILs is proposed as a definitive alternative not only to conventional catalysts used in organic synthesis but also to their corresponding monocationic ILs.

We think that more extensive use of DILs as catalysts in organic reactions could improve not only the catalytic activity and the ease in catalyst recovery and reuse, but also the environmental issue. In fact, DILs seem to be less harmful than the corresponding monocationic ILs and a careful choice of substituents could greatly lower their toxicity [2]. Moreover, a theoretical study to identify the nature of suitable cation substituents and chain length of the spacer for a particular application should be always carried out in order to obtain good experimental results in this field, in both the catalytic performances and in the eco-compatibility field.

It has to be underlined that, to the best of our knowledge, only two papers reported the use of imidazolium DILs as precursors of the corresponding N-heterocyclic carbenes (NHCs), widely used as organocatalysts [7,15]. In this regard, only the syntheses of polydentate NHC ligands derived from DILs for transition-metal catalysis has been extensively studied [56]. We think that this underexplored field should be the subject of further studies in order to test DILs potentiality in this area.

## Figures and Tables

**Table 1:** Reactions of imidazolium-based DILs as organocatalysts.

DILs	Reaction	Products	References
*N*-methyl imidazolium-based DILs with Cl^−^, NO_3_^−^ and PF_6_^−^ anions	Strecker reaction	α-aminonitriles	Verma et al. 2021 [24]
	Carbonylation of amines	carbamates	Verma et al. 2021 [25]
*N*-methyl, butyl, hexyl imidazolium-based DILs bromides, *N*-methyl imidazolium-based DILs carboxylate and acetate	Cycloaddition of CO_2_ to epichlorohydrin	4-(chloromethyl)-1,3-dioxolan-2-one	Guglielmero et al. 2019 [30]
BrØnsted acidic DILs	Esterification of long-chain free fatty acids	Methanol ester of oleic acid	Yang et al. 2019 [31]
	Knoevenagel condensation and multicomponent reaction	5-arylidene barbituric acids and pyrano[2,3-*d*] pyrimidinone derivatives	Daneshvar et al. 2018 [34]
Acidic imidazolium-based DILs with HSO_4_^−^ as counterion	Esterification reaction of phtalic anhydride with different types of alcohols	Phthalates	Zekri et al. 2016 [32]
Acidic heteropolyanion-based DILs	transesterification reaction of methyl acetate with isooctyl alcohol	2-ethylhexyl acetate and methanol	Liu et al. 2018 [33]
*N*-methyl imidazolium-based DILs with Cl^−^ and HSO_4_^−^ anions	Knoevenagel condensation and multicomponent reaction	5-arylidene (thio)barbituric acids, pyrano[2,3-*d*] pyrimidinone derivatives, 2-arylidene malononitriles, 4*H*-pyrans	Sharifi et al. 2019 [35]
*N*-methyl imidazolium-based DILs with Cl^−^, Br^−^ and I^−^ anions	Polymerization of diphenyl carbonate and isosorbide	Polycarbonates	Wang et al. 2021 [36]
*N*-methyl imidazolium-based DILs bromide	One-pot cyclocondensation	3-substituted phenyl-2-(4-(tetrazolo[1,5-*a*]quinolin-4-ylmethoxy)phenyl)thiazolidin-4-ones	Deshmukh et al. 2019 [37]
Graphene oxide supported -SO_3_H functionalized imidazolium-based DIL	One-pot multicomponent reactions	1-carbamatoalkyl-2-naphthols derivatives, spiro[indoline-3,9′-xanthene]trione derivatives, spiro[chromene-4,3′-indoline]-3-carbonitrile derivatives	Patel et al. 2019 [41]
			Patel et al. 2021 [42]
Imidazolium-based DIL catalyst stabilized on Fe_3_O_4_ and SiO_2_ magnetic nanoparticles with MnCl_4_^2−^ as anions	One-pot three-component condensation	4*H*-chromene derivatives	Hormozinezhad et al. 2018 [43]
Brønsted acidic DIL immobilized on silica-coated iron oxide support nanoparticles	One-pot condensation	Bis-pyrazole-5-ols	Rezaei et al. 2020 [44]
Cyclodextrins incorporated with imidazolium-based DIL and supported on silica gel	Hantzsch reaction	1,4-dihydropyridine and polyhydroquinoline derivatives	Moheiseni et al. 2019 [45]
Lignosulfonate/DIL composite	Glaser heterocoupling reaction	Unsymmetrical 1,3-diynes	Lai et al. 2018 [46]
Supported DIL phosphovanadomolybdate anions on mesoporous aluminosilicate	Oxidation of benzene	Phenol	Taheri et al. 2019 [47]
Immobilized imidazolium-based DIL bromide on metal–organic frameworks chromium-benzenedicarboxylate	CO_2_ cycloaddition to epoxides	Cyclic carbonates	Jiang et al. 2021 [48]
Chitosan/DIL composite			Taheri et al. 2018 [49]
Brønsted acidic DIL linked with polyethylene glycol	Conversion of fructose	5-hydroxymethyl furfural	Gogoi and Borah 2018 [50]
ZSM-5 zeolite/DIL			Prasad et al. 2021 [53]
PEG-DIL-based MnCl_4_^2−^	Phase transfer catalyst for nucleophilic substitution of aryl halides	Benzyl azides and thiocyanates	Goodajdar et al. 2019 [54]
Polyethylene glycol methacrylate-grafted imidazolium-based DIL	Multicomponent reaction	*N*-methyl-2-nitro-aryl-benzo[4,5]imidazo[1,2-a]pyrimidine amines	Reddy et al. 2017 [55]

## Data Availability

Not applicable.
